# Death

**Published:** 1887-04-23

**Authors:** 


### WORDS OF CONSOLATION.

[Communications for this column should be addressed, "Chaplain," care of the Editor of THE HOSPITAL, who will gratefully welcome any suggestions or inquiries from matrons, nurses, and the staff generally.]

### XXX.—DEATH.

THERE are two classes of people who have no fear of death. The first are true Christians, whose faith is so lively, and whose love is so warm, that they thoroughly understand their position as strangers and pilgrims on the earth. They not merely confess their brief portion here (as so many do, who are all the while wedded to the world, and would not leave it on any account, so long as they have a straw to cling to), but they are willing, or even glad to resign the present life at any moment, if called to do so. Their life is "hid with Christ in God"; therefore they are sure that "to die is gain." Some of these, when laid on a bed of sickness, will tell you that they would much prefer being taken if God wills. They are evidently prepared to welcome death just as readily, or more so than a night's refreshing sleep. The only thing which ever hinders them from praying for death, is perhaps the thought of their friends, families, or little children unprovided for. They know that the wrench must be terrible to bereaved ones left behind. So although they prefer to depart for their own sakes, they are in a strait (like St. Paul) betwixt two desires, and scarce know which to express in their prayers.

The other class includes some of the very ignorant; but quite as often people who have rejected salvation and become hardened in sin, blinded by unbelief, and therefore utterly careless about a future state, even if they do not disbelieve in it altogether, and take mankind to be worth no more than the beasts that perish. Some of the Communists and Atheists who were executed a few years ago in Paris, were terrible instances of this. They had no fear of death. Several of them, before they were led to execution, openly disavowed their belief in God. Materialists they had lived, Materialists they said they would die. They betrayed no symptoms of fear, because they believed in no resurrection, and certainly not in any future retribution.

But between these two extremes, between the brightest and best of faith and utter unbelief, between the strongest convictions of pardon and grace and complete renunciation of God and Christ, there lies a large majority, living in more or less fear of death. Some fear it rightly, because they are not convinced of pardon, and know that they have no right to be, so long as sin remaineth at their door. Some fear it because they think that the illness, or accident, which ends their life must needs involve more pain than any other. Some fear it because they have heard and read a good deal about death, and are therefore convinced that it must be a very dreadful thing. Others, again, fear it; they cannot tell you why. Analyse their feelings, and you may find they have no fair reason for doubting forgiveness; they do not shrink from pain; they do not believe all they hear or read about death; they think its terrors must be exaggerated in many minds, and yet (they hardly know why) they are not free from those terrors themselves; they cannot quite grasp the truth of their Saviour's words, "It is not death, but sleeping."

We have more to say to some of our readers who may feel themselves to form part of this majority we have attempted to describe. Meanwhile, we would earnestly beg them not to shirk a question of such awful importance.

(*To be continued.*)

THE GUILD OF ST. BARNABAS FOR NURSES.—This Guild has been established for many years, and has branches in Liverpool, Manchester, Worcester, Gloucester, and Leamington. The chaplain, Rev. C. F. Russell, 35, Brook Street, Holborn, will supply any information.

